# Drug-Resistance Mechanisms in *Vibrio cholerae* O1 Outbreak Strain, Haiti, 2010

**DOI:** 10.3201/eid1711.110720

**Published:** 2011-11

**Authors:** Maria Sjölund-Karlsson, Aleisha Reimer, Jason P. Folster, Matthew Walker, Georges Anicet Dahourou, Dhwani Govil Batra, Irene Martin, Kevin Joyce, Michele B. Parsons, Jacques Boncy, Jean M. Whichard, Matthew W. Gilmour

**Affiliations:** Centers for Disease Control and Prevention, Atlanta, Georgia, USA (M. Sjölund-Karlsson, D.G. Batra, K. Joyce, M.B. Parsons, J.M. Whichard); Public Health Agency of Canada, Winnipeg, Manitoba, Canada (A. Reimer, M. Walker, I. Martin, M.W. Gilmour); IHRC Inc., Atlanta (J.P. Folster); Centers for Disease Control and Prevention, Port-au-Prince, Haiti (G.A. Dahourou); Ministry of Public Health and Population, Port-au-Prince (J. Boncy)

**Keywords:** Vibrio cholerae, cholera, antimicrobial drug resistance, bacteria, Haiti, outbreak, strain, O1

## Abstract

To increase understanding of drug-resistant *Vibrio cholerae,* we studied selected molecular mechanisms of antimicrobial drug resistance in the 2010 Haiti *V. cholerae* outbreak strain. Most resistance resulted from acquired genes located on an integrating conjugative element showing high homology to an integrating conjugative element identified in a *V. cholerae* isolate from India.

*Vibrio cholerae* is the bacterium that causes cholera, a disease characterized by acute watery diarrhea, vomiting, muscle cramps, and severe dehydration (*1*). The bacterium has many serogroups, but only toxin-producing serogroups O1 and O139 cause epidemic cholera. The primary treatment for cholera is rehydration with oral or intravenous fluids (*2*). For severe cases, antimicrobial agents may reduce the volume and duration of diarrhea (*1*,*2*). Tetracyclines (e.g., doxycycline), fluoroquinolones (e.g., ciprofloxacin), macrolides (e.g., erythromycin), and trimethoprim/sulfamethoxazole have commonly been used to treat cholera (*2*).

Antimicrobial drug resistance can undermine the success of antimicrobial therapy. Several reports have documented tetracycline- and fluoroquinolone-resistant *V. cholerae*, and multidrug resistance is increasing (*3*). Antimicrobial drug resistance in *Vibrio* spp. can develop through mutation or through acquisition of resistance genes on mobile genetic elements, such as plasmids, transposons, integrons, and integrating conjugative elements (ICEs). ICEs integrate and replicate with the host chromosome and can excise themselves and transfer between bacteria by conjugation (*4*). ICEs commonly carry several antimicrobial drug resistance genes and play a major role in the spread of antimicrobial drug resistance in *V. cholerae* (*5*). The first *V. cholerae* ICE described was in an O139 isolate in Madras, India, in 1992 and was named SXT after the resistance phenotype it conferred (trimethoprim/sulfamethoxazole) (*6*). Many O139 and O1 isolates have since acquired SXT or a closely related ICE (*4*,*5*).

We describe antimicrobial drug resistance mechanisms in the 2010 Haiti *V. cholerae* O1 outbreak strain. Most of the resistance is caused by acquired genes located on an ICE with high similarity to an ICE identified in a *V. cholerae* O1 isolated in India.

## The Study

During October 2010–January 2011, a total of 122 clinical isolates of laboratory-confirmed *V. cholerae* O1 were recovered by the National Public Health Laboratory in Haiti and submitted to the Centers for Disease Control and Prevention (CDC; Atlanta, GA, USA) for characterization. Disk-diffusion antimicrobial drug susceptibility testing was performed at the National Public Health Laboratory and CDC. MICs were determined by broth microdilution at CDC by using Sensititer plates (CAMPY and CMV1AGNF; Trek Diagnostics, Cleveland, OH, USA) according to the manufacturer’s instructions with the following modifications: Mueller-Hinton broth without blood was used on the CAMPY plate, and for both plates, a final inoculum concentration of 5 × 10^4^ to 5 × 10^5^ CFU/mL was targeted. *Escherichia coli* American Type Culture Collection (ATCC; Manassas, VA, USA) 25922, *Staphylococcus aureus* ATCC 29213, *Enterococcus faecalis* ATCC 29212, and *Pseudomonas aeruginosa* ATCC 27853 were used for quality control testing. Where available, Clinical and Laboratory Standards Institute criteria specific for *V. cholerae* were used (*7*). For drugs lacking such criteria, manufacturers’ criteria, Clinical and Laboratory Standards Institute criteria for *Enterobacteriaceae,* or consensus breakpoints used by the National Antimicrobial Resistance Monitoring System were applied (*8*,*9*). Furazolidone was tested only by disk diffusion, and azithromycin was tested only by broth microdilution.

Results for all 122 outbreak isolates were similar. They showed susceptibility to azithromycin and tetracycline, reduced susceptibility to ciprofloxacin (MIC 0.25–1.0 mg/L), and resistance to furazolidone, nalidixic acid, sulfisoxazole, streptomycin, and trimethoprim/sulfamethoxazole.

With a common susceptibility pattern among all outbreak isolates, 1 isolate, 2010EL-1786 (deposited under ATCC BAA-2163), was chosen for molecular characterization. PCR was used to screen the isolate for the following resistance genes: *strA*, *strB*, *sul1*, *sul2*, *dfrA1*, *dfrA10*, and *dfrA12* (*10*). In addition, the *gyrA* and *parC* genes were sequenced to identify quinolone resistance–determining region mutations. PCR was performed according to standard protocols by using the HotStarTaq PCR Master Mix (QIAGEN, Valencia, CA, USA). DNA sequencing was performed by using a 3730 DNA Analyzer (Applied Biosystems, Foster City, CA, USA).

The isolate 2010EL-1786 contained *strA/B*, *sul2*, and *dfrA1*, which mediate resistance to streptomycin, sulfisoxazole/sulfamethoxazole, and trimethoprim, respectively. Nalidixic acid resistance and decreased susceptibility to ciprofloxacin were attributed to mutations in *gyrA* (Ser83Ile) and *parC* (Ser85Leu). The mechanism responsible for furazolidone resistance was not identified. Mutations in the *nfsA* and *nfsB* genes are associated with furazolidone resistance in *E. coli,* but inspection of the 2010EL-1786 sequence failed to identify these genes.

Location of the resistance genes was analyzed by whole-genome sequencing of 2010EL-1786. Single-end 454 pyrosequencing (GS FLX-Titanium; Roche Diagnostics, Indianapolis, IN, USA) reads and single-end 36-bp Illumina Solexa (GAIIe; Illumina, San Diego, CA, USA) reads were assembled de novo by using Newbler (Roche Diagnostics) and CLC Genomics Workbench (CLC bio, Cambridge, MA, USA) software. Sequence finishing was performed by using Sanger sequencing of fosmid clones (*11*).

**Figure 1:**
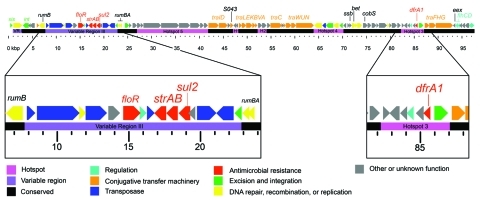
Genetic organization of the 2010 Haiti *Vibrio cholerae* O1 integrating conjugative element (ICE), ICE*Vch*Hai1. The ICE contained 97,915 bp and 95 open reading frames. Coding sequences were identified and manually annotated by using an in-house modified version of GenDBv2.2 (Center for Biotechnology at Bielefeld University, Bielefeld, Germany, www.cebitec.uni-bielefeld.de/groups/brf/software/gendb_info/index.html). Regions conserved among previously sequenced ICEs are indicated in black, regions of variability in purple, and previously identified hotspots of homologous recombination in pink. Conserved genes involved in conjugation are indicated in orange. Genes associated with antimicrobial drug resistance (*floR* [chloramphenicol], *strAB* [streptomycin], *sul2* [sulfamethoxazole], and *dfrA1* [trimethoprim]) are indicated in red. The complete sequence of ICE*Vch*Hai1 has been deposited into GenBank under accession no. JN648379.

Whole-genome sequencing identified an ICE inserted in the *prfC* gene. This ICE, designated ICE*Vch*Hai1, was 97.9 kb and contained 95 open reading frames (Figure 1). All resistance genes identified were located on ICE*Vch*Hai1. In addition, *floR*, a chloramphenicol resistance gene, was detected. The *strA*, *strB*, *sul2*, and *floR* genes were part of an ≈17-kb fragment inserted into the *rumB* gene, whereas the *dfrA1* gene was located ≈70 kb further downstream. Whole-genome sequencing also indicated a chloramphenicol acetyltransferase gene, *catB9*, that was not part of the ICE.

**Figure 2:**
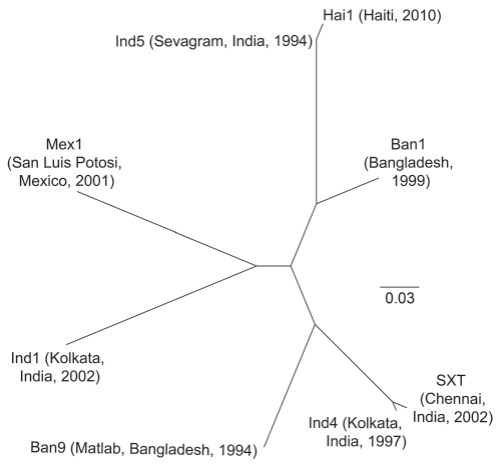
Phylogenetic tree illustrating the genetic relatedness between the Haiti integrating conjugative element (ICE) ICE*Vch*Hai1 and other ICEs described in *Vibrio cholerae* (ICE*Vch*Ban1, ICE*Vch*Ban9, ICE*Vch*Ind1, ICE*Vch*Ind4, ICE*Vch*Ind5, ICE*Vch*Mex1, and SXT*).* Each ICE is listed by an abbreviated name followed by geographic origin and isolation year of the isolate in this analysis. The sequence of ICE*Vch*Hai1 was aligned with the other *V. cholerae* ICE sequences by using the software Progressive Mauve (http://asap.ahabs.wisc.edu/mauve/download.php), and a neighbor-joining phylogenetic tree was constructed by using PHYLIP (PHYLIP [Phylogeny Inference Package] version 3.69; distributed by J. Felsenstein, Department of Genome Sciences, University of Washington, Seattle, WA, USA). Branch lengths indicate the genetic distance between the different ICEs. The ICE*Vch*Hai1 showed highest homology (5 single-nucleotide difference) to ICE*Vch*Ind5, an ICE first detected in an isolate of *V. cholerae* O1 in Sevagram, India, in 1994. The ICE sequences in the analysis can be accessed by using the following GenBank accession nos.: Ban1, GQ463139; Ind1, GQ463144; Ind4, GQ463141; Ind5, GQ463142; Mex1, GQ463143; Ban9, CP001485; and SXT, AY055428. The complete sequence of the ICE*Vch*Hai1 has been deposited into GenBank under accession no. JN648379. Scale bar indicates nucleotide substitutions per site.

The genetic relatedness of ICE*Vch*Hai1 was assessed by comparison with 7 other ICE sequences (*4*). Sequence alignments were performed by using Progressive Mauve (http://asap.ahabs.wisc.edu/mauve/download.php) and visualized with PHYLIP version 3.69 (distributed by J. Felsenstein, Department of Genome Sciences, University of Washington, Seattle, WA, USA). ICE*Vch*Hai1 showed highest homology to ICE*Vch*Ind5, an ICE derived from a *V. cholerae* isolate from India (Figure 2). These ICEs differed by only 5 single-nucleotide polymorphisms.

## Conclusions

In October 2010, an epidemic caused by toxigenic *V. cholerae* O1, serotype Ogawa, biotype El Tor strain, was reported from Haiti. We confirmed that the outbreak strain was multidrug resistant and displayed resistance to furazolidone, nalidixic acid, sulfisoxazole, streptomycin, and trimethoprim/sulfamethoxazole and decreased susceptibility to ciprofloxacin. Genetic mechanisms responsible for resistance to 5 of these drugs were identified. Sequencing also detected *floR*, a gene commonly associated with chloramphenicol resistance in *Enterobacteriaceae* (MICs >32 mg/L) (*12*). However, in this study, *floR* was not associated with resistance; isolates from Haiti displayed chloramphenicol MICs of 4–16 mg/L (*7*). Clinical non-Haiti isolates lacking the *floR* gene displayed MICs <1 mg/L. Why the *floR* gene did not confer resistance in *V. cholerae* remains to be investigated but might be because of lower expression levels. Sequencing also showed a chloramphenicol acetyltransferase gene, *catB9*. How this gene affects chloramphenicol MICs in *V. cholerae* remains to be determined.

Most of the acquired resistance genes were located on an ≈97-kbp ICE termed ICE*Vch*Hai1. The presence of an ICE in *V. cholerae* from Haiti was documented by Chin et al. in 2011 (*13*). ICE*Vch*Hai1 showed high homology to ICE*Vch*Ind5, an ICE first identified in a *V. cholerae* isolate from Sevagram, India, in 1994. Since then, ICE*Vch*Ind5 has persisted among clinical isolates in India; a recent study of O1 strains isolated in India during 1994–2005 confirmed that ICE*Vch*Ind5 was the only ICE that persisted during the study period (*14*).

Drug-resistant *V. cholerae* is a global health concern because resulting infections can be more severe and difficult to treat. Infections with drug-resistant *V. cholerae* can result in higher case-fatality rates, prolonged hospitalizations, more secondary infections, and increased health care costs. During an outbreak in Guinea-Bissau, case-fatality rates increased from 1% to 5.3% after the outbreak strain acquired multidrug resistance (*15*). To limit development and spread of antimicrobial drug resistance among *V. cholerae*, treatment with antimicrobial agents should be restricted to patients with severe dehydration or other conditions that truly warrant their use. Surveillance should continue for antimicrobial drug resistance among *V. cholerae* isolates from Haiti.
